# Experiences From Developing and Upgrading a Web-Based Surveillance System for Malaria Elimination in Cambodia

**DOI:** 10.2196/publichealth.6942

**Published:** 2017-06-14

**Authors:** Clementine Fu, Sérgio Lopes, Steve Mellor, Siddhi Aryal, Siv Sovannaroth, Arantxa Roca-Feltrer

**Affiliations:** ^1^ Malaria Consortium Cambodia Phnom Penh Cambodia; ^2^ Independent Consultant Phnom Penh Cambodia; ^3^ Malaria Consortium Asia Regional Office Bangkok Thailand; ^4^ The National Center for Parasitology, Entomology and Malaria Control Phnom Penh Cambodia; ^5^ Malaria Consortium Global Office London United Kingdom

**Keywords:** malaria elimination, surveillance system, Cambodia

## Abstract

Strengthening the surveillance component is key toward achieving country-wide malaria elimination in Cambodia. A Web-based upgraded malaria information system (MIS) was deemed to essentially act as the central component for surveillance strengthening. New functionality (eg, data visualization) and operational (eg, data quality) attributes of the system received particular attention. However, building from the lessons learned in previous systems’ developments, other aspects unique to Cambodia were considered to be equally important; for instance, feasibility issues, particularly at the field level (eg, user acceptability at various health levels), and sustainability needs (eg, long-term system flexibility). The Cambodian process of identifying the essential changes and critical attributes for this new information system can provide a model for other countries at various stages of the disease control and elimination continuum. Sharing these experiences not only facilitates the establishment of “best practices” but also accelerates global and regional malaria elimination efforts. In this article, Cambodia’s experience in developing and upgrading its MIS to remain responsive to country-specific needs demonstrates the necessity for considering functionality, operationalization, feasibility, and sustainability of an information system in the context of malaria elimination.

## Introduction

Within the Greater Mekong Subregion (GMS), Cambodia has seen dramatic shifts in malaria incidence during the last 10-15 years. Since 2000, confirmed malaria cases have decreased by over 75%, partly as a result of targeted treatment and management activities outlined in the National Strategic Plan 2011-2025 [1,2]. Accordingly, in line with the regional shift toward malaria elimination and the country’s waning malaria burden, an update to this plan was developed in 2015—the malaria elimination action framework (MEAF) 2016-2020 [3]. This framework sets out an ambitious plan to eliminate malaria by 2025, with a particular emphasis on the surveillance system and accompanying activities. It reflects new strategic updates based on changes in the country’s epidemiological and programmatic contexts, while taking into account recommendations from previous containment experiences and other regional and global policy guidelines. Strengthening the surveillance component is a necessary marker toward achieving country-wide elimination. Consequently, considering the need to incorporate and facilitate the capture of elimination-focused data, a new and upgraded information system was deemed essential to act as the central component for the surveillance plans. The Cambodian experience of identifying the essential changes and critical attributes for this new information system can provide a model for other countries at various stages of the disease control and elimination continuum. In this viewpoint paper, we highlight the main steps needed to ensure a smooth and progressive shift from an offline information system toward an integrated Web-based system.

## Cambodia Malaria Information System in Cambodia: A System Upgrade to Meet Elimination Needs

The first national malaria information system (MIS) was constructed as a Microsoft Access database (Microsoft Corporation, Redmond, USA) in 2009. It aimed to collect and analyze data reported from various levels of the health system to the centrally based National Centre for Parasitology, Entomology, and Malaria Control (CNM) [4].

The MIS evolved over time, and by the end of 2013, several modules and functionalities existed, namely, (1) individual case-based line listing reported from village malaria workers (VMWs), district-level health facilities (HFs), and private providers; (2) provincial and national data reports, including epidemiological summaries and basic visual outputs; and (3) rudimentary data flow for real-time reporting mechanisms.

### Cambodia Malaria Information System—Initial Development, Objectives, and Functionalities

The system was originally developed to primarily capture monthly individual-level malaria case data from both the national community-level VMW program and government-supported health facilities in malaria-endemic provinces. The prototype system also added a level of granularity (eg, geographical and functional detail) deemed necessary to shift toward elimination [5]. Such level of detail is not present in the national Ministry of Health (MOH)-sponsored health management information system (HMIS) database, which only captures aggregate case data at the provincial and district level.

The MIS has since expanded to also capture data directly from private providers. Further functionalities were also added to the MIS to support and document other CNM-related malaria activities, including intervention coverage like bed net distribution data.

Other components were created as operational and analytical needs arose; specifically, efforts to analyze data at the village level for risk stratification purposes resulted in a new, separate module [6].

Additionally, a quarterly malaria bulletin was introduced to improve feedback and promote data-driven decisions at both peripheral and central levels.

### Initial Real-Time Case Reporting Activities

During the initial development phase of the MIS, an attempt at utilizing the system for real-time reporting activities was implemented in the form of day 0 or day 3 SMS (short text message, SMS) alert systems [4,6]. The day 3 system was created to notify relevant health facility or operational district (OD) officials about post-day 3 parasitaemic patients, indicating the potential need for an active response. This system was then later expanded to include day 0 case notifications, allowing for further field level responses. A separate server was set up to capture this SMS information, which was then pushed passively into the MIS. Hence, although the MIS could receive and gather this data immediately, it did not have the ability to process the information and communicate directly back to the SMS server. At that time, since no operational field follow-up was done that required supplementary SMS data entry from other sources (eg, health facility report for interventional treatment of an initially reported day 3 positive case), the MIS structure did not need to have the interoperability and flexibility needed for an active response.

### Malaria Information System (MIS) Usage and Data Flow

**Figure 1:**
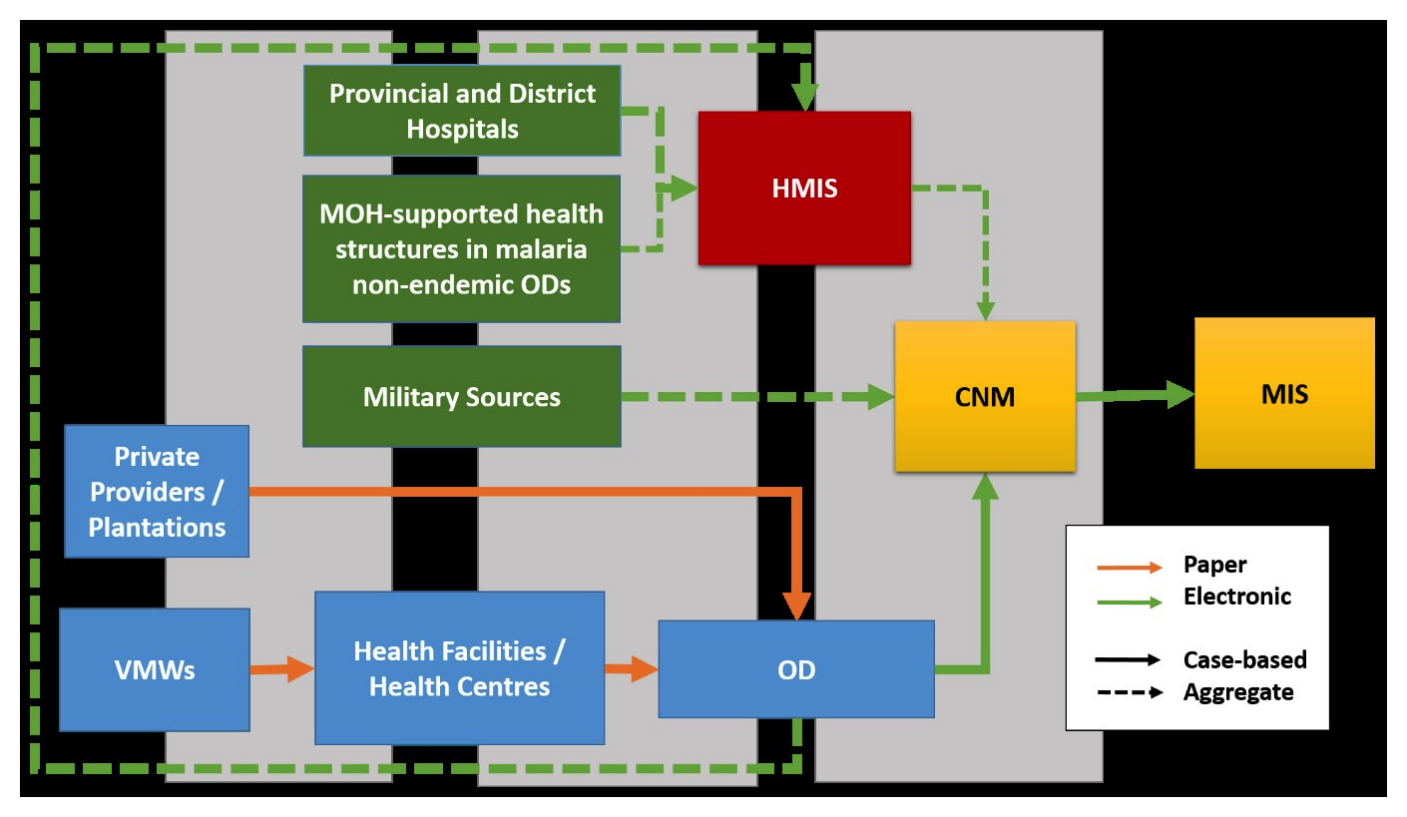
Malaria surveillance data flow into MIS (Initial phase).

All cases (tested, confirmed, and treated) localized to the village level are recorded via paper-based forms from VMWs, health facilities or health centers, and private providers (public private mix) in CNM-supported (malaria-endemic) districts. This data is collated monthly by each source’s respective OD and then entered electronically into the MIS Access file to be sent to CNM and compiled into the comprehensive database (see Figure 1). Data from all MOH-maintained health facilities throughout the country is also entered in aggregate form to the HMIS. As a result, the lowest level of localization for HMIS-captured data is at the health facility level. Data from the HMIS is extracted regularly by CNM and entered manually into the MIS. For those areas not captured by the MIS directly (eg, district and provincial hospitals, and nonendemic regions), the data helps to complete the full national malaria context. For areas already covered by the MIS, a cross-checking analysis is done to monitor the comparative data quality. Accordingly, end users of the full MIS are located at the OD and central level only.

Data flow mimics ground-level operations, functioning primarily in a uni-directional manner toward the central level without a specific operational response. This conceptually embodies the classical model of a malaria surveillance system [5]. The mixed paper-based and electronic reporting system is still currently in use until a full transition to electronic reporting is piloted, evaluated, and fully operational.

### The Need for Upgrade—New Nationally Integrated Surveillance and Elimination Activities

According to the malaria elimination action framework 2016-2020 strategy [3], Cambodia’s plans for elimination include a phased approach wherein elimination-targeted ODs are planned to scale up progressively from 18 in 2016 to 42 in 2018. This categorization is based on individual districts’ risk stratification.

As this phased approach takes place, new and accompanying activities, including reactive case investigations, proactive detection, and real-time responses, become crucial components to a successful elimination strategy [7].

Functionally, the MIS needs to respond to an implementation plan with different ODs in different risk strata and needs to be able to process incoming data from multiple sources, activities, and geographical regions. The necessity for mobile platform integration is also critical, as technological tools have been proven to effectively facilitate field-level elimination responses [8].

### System Components Necessary for Elimination

Defining the key features of the system in a participatory way is essential prior to the development and roll out of any surveillance system [5,9]. Toward that end, CNM and its key technical partners agreed upon several critical data elements, indicators, and surveillance activities for malaria elimination through a series of working groups. The corresponding components needed in the complementary information system mirror the main points agreed in this group (Ministry of Health, Kingdom of Cambodia, Surveillance for Elimination: Operational Manual, unpublished data). The necessity for integrated and interrelated data justifies the transition to other type of platforms and database structure responsive to these requirements.

#### Real-Time Reporting and Case Notification

As caseload continues to drop, the need to respond to every case quickly for appropriate treatment and follow-up becomes more critical. Ideally, every positive case reported from an elimination OD must be registered in real time, regardless of which point of care source it originated at [10]. Therefore, all sources must have a direct notification link (eg, mobile phone, SMS, and so on) to the central information system, allowing the subsequent outwards data flow to occur. This communication is a critical piece of the elimination strategy, especially considering the potential mobility and nonpermanent status of migrant populations at highest risk for malaria.

#### Case Investigation

Once a case is reported, an investigation should be initiated in a timely manner to obtain further patient information, establish transmission potential, and determine need for additional response activities [11]. Operationally, the investigation may involve environmental inspection and in-depth travel history questions to be captured on a mobile platform. Additional activities or interventions may be recommended, either based on the team’s manual assessment or an automatic criteria-defined trigger from the central database. From a systems perspective, this also suggests that an initial case captured by the database from a particular source will need to be linked to additional follow-up information from a different source.

#### Foci Investigation

In elimination settings, additional follow-up on a passively detected index case is insufficient; foci investigation is necessary to determine where the infection occurred, whether active vectors may be present to transmit the parasite, and potentially identify geographical centers of interest [7,11]. Based on results from the initial case investigation, an operational decision can be made about the need for foci information. This data can additionally inform geographical risk and vector surveillance initiatives. Although it is sufficient for foci information in the information system to be linked to the case through its reporting source, additional structural considerations are necessary to analyze risk and interventions.

#### Case Response and Intervention Coverage

Once the case investigation is complete, additional measures may be taken at the point of identification. Reactive case detection (RACD) around the index case and intervention coverage may be necessary, and need to be documented as a result of the initial case notification [7]. These activities can also be mapped and identified via geolocation. The operational response may be different for each case, as determined by multiple factors (eg, the OD in which the case is located, the village-level risk, and so on)—therefore, the system should be able to automatically identify the defined course of action based on captured information and trigger resultant notifications to the responsible field-level actors.

#### Risk Stratification

Initial efforts at stratifying malaria risk at the village level proved to be useful for directing interventions [6] However, this exercise was only partially automated—the risk indicator was limited and could not incorporate other nonincidence related data. Additionally, the analyses suffered from lack of data completeness and obligatory usage of time-delayed malaria data, decreasing the practical benefits. Currently, under the elimination context, a more fine-grained classification is necessary to guide the classification and transition of new MEAF risk categories. Regular analyses will assist field-level facilities to direct intervention and financial coverage [5,10]. Both monthly passive data from burden reduction areas and real-time data from elimination areas can be combined with other key factors to create a consistently updated picture of malaria risk within the country. The core system therefore needs to be able to actively interact with and integrate both types of data to create an automated, systematic output.

#### Data Visualization

Quick, regularly updated data visualization and utilization is a key component to decentralizing malaria control and elimination efforts [5,9,10,12]. Provincial and field-level staff can benefit from understanding and interpreting infection trends to better direct their resources. In the Access system, although the OD users could produce reports based on their own district-level data, the full scope of updated data is only captured and stored locally at the central level. Data usage is purely passive through the production of quarterly dashboards. For field staff, data application must be useful, immediate, and precise; indicators and visualizations should be predefined as much as possible to facilitate ease of operations. Bidirectionality of data flow is critical to empower those at points of care to understand the importance of their role in the surveillance system and take more ownership in these processes [5,8,13].

## Moving Toward a Web-Based Malaria Information System: Key Requirements

By 2011, all 45 malaria-targeted ODs had received training and incorporated the original MIS, evidencing the rapid uptake and strong ownership of the system by the national program [6].

As Cambodia approached national and regional goals for malaria elimination, new functionality development (eg, data visualization) and operational aspects (eg, data quality) were the primary broad issues for improving the information system. However, other context-specific aspects are equally important in defining the framework, particularly system acceptability and sustainability [14] For instance, feasibility issues at the field level (eg, user acceptability at various health levels) and sustainability needs (eg, long-term system flexibility) were considered key points to be addressed.

### Corresponding System Development Timeline With Malaria Elimination Action Framework (MEAF) Elimination Plans

To respond to the ambitious phasing and objectives set by the MEAF, not only is it necessary to improve or reconstruct the current passive reporting system, but also to develop and roll out all newly required malaria elimination modules. Thus, it is crucial to follow a phased approach to ensure that each component can operate successfully both in isolation and in coordination with the entire system.

The first key step was to develop and transition to a Web-based platform. This new system, completed and piloted in 2016, moves passive data reporting from a local database to a centrally accessible server. Not only will this change facilitate speed of data entry and immediate feedback to the field level, but it also expands the necessary framework for subsequent development (see Table 1). In the following phase, previously described malaria elimination modules will be added to the MIS in a layered manner, ensuring sufficient flexibility to account for further additions or changes identified during the implementation phase (see Mutilmedia Appendix 1).

**Table 1:** Characteristics of local versus Web-based malaria information system (MIS) in Cambodia.

Characteristics	Microsoft Access: local-based MIS^a^	Web-based MIS
Modality	Offline—Fill in and send to CNM^b^ monthly	Online—Web-based, “client-server”
Access	Local—Software and app must be downloaded on each device	Web—Accessible from any device with Internet access
Users	OD^c^, CNM Smaller number of users enabled	OD, CNM, HF^d^, HCs^e^, VMW^f^ Can handle many users
Importation	Manual from HMIS^g^ Cannot be automated	Has capacity to be automated from HMIS and other individual databases (eg, private providers)
Size	2GB maximum	10 GB+
Other	Limited capacity for capturing and communicating with mobile app data	Greater flexibility for data input and outputs from other sources, including mobile

^a^MIS: malaria information system.

^b^CNM: National Centre for Parasitology, Entomology, and Malaria Control.

^c^OD: operational district.

^d^HF: health facility.

^e^HC: health center.

^f^VMW: village malaria workers.

^g^HMIS: health management information system.

Despite the time pressure to roll out elimination activities (18 ODs were targeted for elimination in 2016), it is essential to guarantee that the system upgrade and implementation is responsive to functional and operational needs. Although the phased process may not be the most rapid approach to reach elimination, the continuous “learning by doing” approach responds to country needs as planning between stakeholders progresses. Considering the added functions of elimination-focused field activities, concentrated technical discussions on the surveillance system are necessary to clearly establish country-specific procedures. Conceptualization and development of the core information system are tied to these decisions, as the technical components directly reflect and respond to surveillance definitions. Additionally, it is crucial to ensure that the system incorporates expansive flexibility to allow for future changes in policy or operation. Despite initial guidance on the key surveillance activities for malaria elimination, discussions about the malaria surveillance system in Cambodia are ongoing and may change based on program prioritization.

### Required Functionalities for MIS Upgrade

#### Functional Attributes: Database Structure and Web Attributes

A significant change in the back-end data structure of the upgraded MIS is crucial to ensure system adaptability and functionality. The relational tables in the original Access-based MIS were constructed by separating data sources, mimicking the functional data flow needs at the time. Case data detected and reported by a VMW were stored independently from case data detected by a health facility, even though individual case-level information captured by each provider was similar. Hence, cases were linked to their respective data sources rather than pooled together as an aggregate collection of cases. However, as the system evolves to respond to elimination needs, a source-based structure is no longer the most efficient solution for data storage and systems processing. A secondary case investigation or case follow-up activity might be completed by a different field source than the initial reporting source. The data captured from all subsequent activities need to be linked to the original report, an effort which would be inefficient and impractical to implement in a source-based structure.

Considering the lack of a country-systematized unique patient identifier, the information system must adopt a case-based structure, wherein all cases are stored in the same table regardless of reporting source. This produces a system-derived case identification, and allows data from all initial and follow-up activities to be linked without data replication and system inefficiency. Each case’s initial reporting source is still recorded to allow for programmatic monitoring. This change in data structure prepares the system to be more responsive in accumulating and analyzing information from elimination activities. Follow-up investigations and interventions can be linked to their initial case reports, enabling capacity to monitor program performance. It also allows for increased ease of query from the system database, eliminating the need to extract information from multiple separate sources. Long-term future possibility also exists for additional data sources or elimination-context activities, changing the types of incoming data and intersecting relationships. A case-based structure enables far greater system flexibility for currently unknown changes.

A main feature of the MIS upgrade is its Web-based functionality. Since the original MIS is a locally stored database, the most updated and complete dataset is only accessible by the CNM central-level data unit. Any additions or changes made at this level are not reflected in field-level office datasets unless newly updated files are sent or the Access package is reinstalled locally. Under that system, regular, timely data visualization and analysis is not possible at the peripheral levels where data is entered; rather, the primary field role is simply to report passively and wait for feedback from the CNM epidemiology unit.

As the country moves toward elimination, immediate data visualization at lower levels of the system is key to ensure appropriate, targeted responses [5,7,8]. With a Web-based functionality, automated dashboards can be produced and integrated within the MIS user interface, enabling rapid up-to-date data visualization at the lower operational levels. The increased operational capacity of a client-server relationship creates greater potential for both the system and users. This system gains an enhanced ability to communicate and acquire data through different platforms, particularly for the usage of Internet-based devices. Intersystem exchanges are also greatly enhanced, particularly for data analysis and feedback.

**Figure 2:**
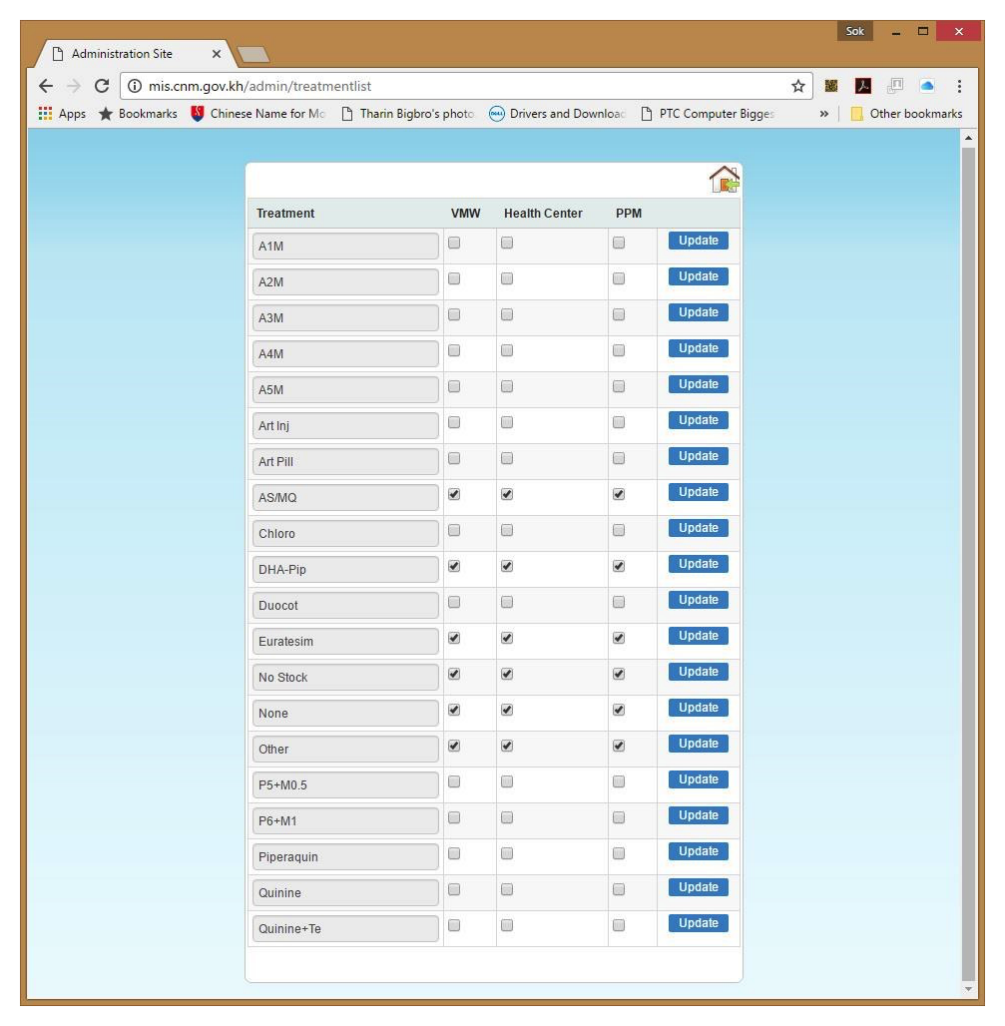
Examples of malaria information system (MIS) attributes and their immediate system effect (treatment guidelines).

Additionally, any changes made to the core data reporting can be reflected immediately—for instance, if treatment guidelines are changed to validate administration of a new drug, CNM can add this option to the central administrative reporting database which updates instantaneously. Field-based end users can view and select the new option during data entry immediately rather than having to spend time and incur resource costs to implement another database configuration (see Figure 2).

Finally, utilizing a Microsoft structured query language (SQL) database rather than an Access one increases the capacity to store larger quantities of data. As elimination activities involve more operational resources and complex data analysis, they will generate more information and require greater systems processing. SQL offers greater flexibility in size for future expansions while still retaining retrospective data from all years of implementation.

**Figure 3:**
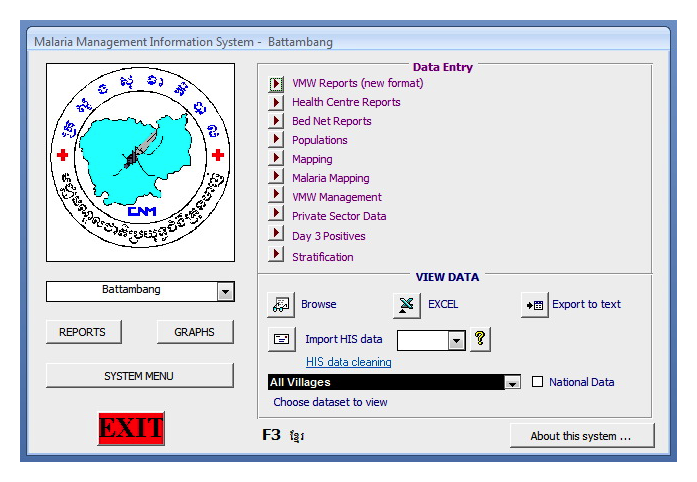
Microsoft Access malaria information system (MIS) main page.

**Figure 4:**
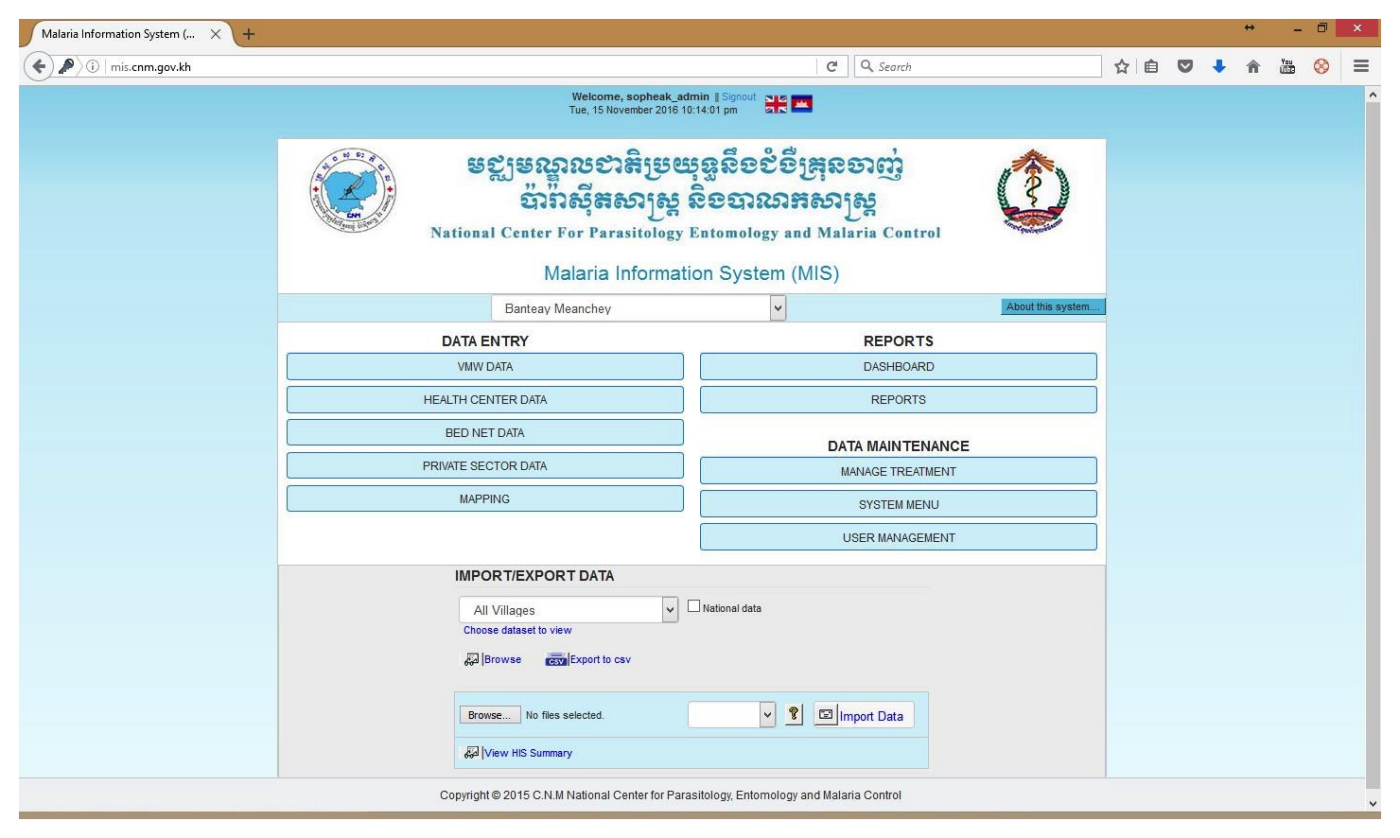
Web-based malaria information system (MIS) main page.

**Figure 5:**
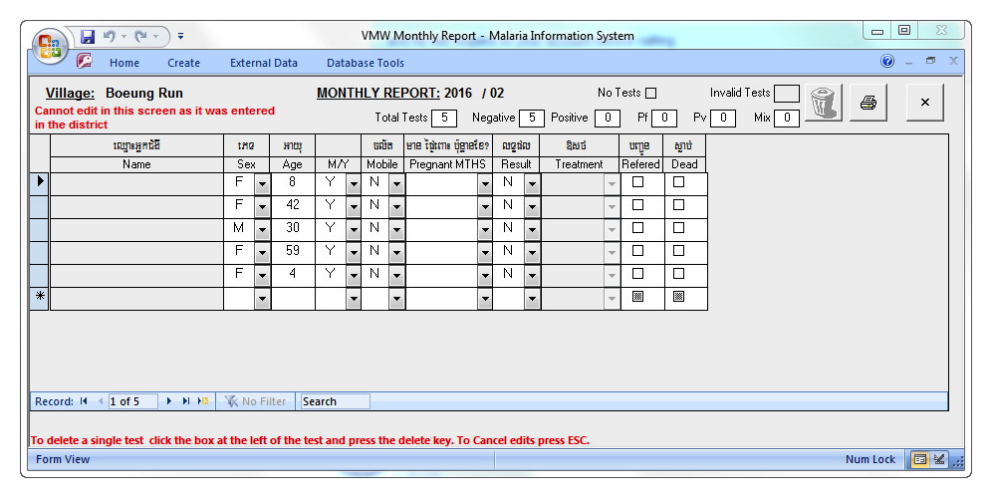
Microsoft Access malaria information system (MIS) village malaria worker (VMW) data entry page.

**Figure 6:**
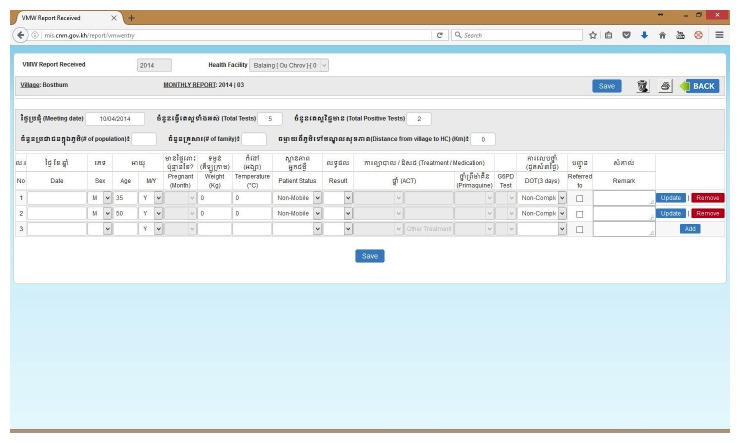
Web-based malaria information system (MIS) village malaria worker (VMW) data entry page.

During the initial stage of the MIS upgrade, it was critical to ensure that the existing Access-based MIS modules and functionalities were replicated for continuity. The user-viewed front end design remained relatively similar to the existing MIS in order to minimize the impact of the system upgrade to its main end users (periphery staff). Data entry and core modules were visually maintained as similar as possible to ease the transition, guarantee maximum user acceptance and adherence to the new system, and minimize training needs (see Figures 3-6).

A new key functionality is the tiered user administration access. All users are assigned a level based on their function within the system (eg, OD, HF, and VMW), each of which accords a different type of functionality and data access. Peripheral users can only view their type’s operational modules and can only enter data for cases which occur in their geographical coverage area. Access to additional functions, including customizable data dashboards, are also determined by the user category. This allows for the system to be developed as a single front-end structure with different viewing permissions, rather than necessitating multiple structures to be built for different user groups. More importantly, the central server platform also allows multiple users to access the same system components, increasing the potential for user efficiency in data entry. This tiered access was designed to be managed by a central level user (CNM) who can administer changes according to the needs.

#### Operational Attributes

##### System Piloting and Feedback

The phased approach used in the MIS development will enable health staff at District and Provincial (PHD) levels to be gradually exposed to the system as it develops. This is particularly important with new or unfamiliar features like data visualization through dashboards. It also encourages system ownership by giving additional access to immediately updated information, supporting decision making at lower administrative units. Initially, OD and PHD staff can become familiarized with simplified dashboards designed to display the most relevant information for activities and implementation. As the system develops and malaria elimination activities are scaled up, the dashboards are expected to integrate more data with greater detail, as well as add customization to understand how their raw data is utilized.

Initial feedback from first-stage pilots at the OD level have already indicated increased levels of systems convenience and ease of usage; additionally, introducing features in stages allows time and response to incorporate suggestions from end users’ experience. Although these pilot activities were not comprehensive, they were intended as low-scale feasibility assessments by actual field representatives to obtain critical practical feedback for further improvements [9].

##### Data Checks and Quality

Ensuring data quality, completeness, and accuracy is critical not only for malaria elimination activities, but also builds crucial aspects of a surveillance system database [5,10]. The enhanced MIS was designed with a built-in capacity to alert and correct data entry errors, reduce potential user mistakes, and improve variable organization. Minimizing data problems at the point of entry reduces central-level time and resources for database cleaning and field monitoring. All modules, including those on mobile platforms have been designed with similar preliminary data quality checks to reduce reporting errors.

The MIS upgrade also automates the capacity to identify missing data or reports depending on the submission timelines defined by CNM. Specific field evaluation indicators are generated for follow-up. Reporting completeness is critical to ensure proper surveillance coverage, and is considered a major priority area for improvement. Previous risk stratification exercises were not automated due to gaps in data completeness, necessitating expensive and time consuming field operations to actively fill them retrospectively through field visits. This functionality is expected to have a wider impact on the system and operations, as it can generate automatic updates to better inform planning, execution, and oversight.

Lastly, the new MIS is expected to automatically integrate data from at least two other non-CNM operated sources (private sector outlets and the national health management information system), as well as potentially others in the future. This regularized data flow within the MIS will not only ease the burden of potentially duplicative data entry from periphery users required to report to multiple sources, but also streamlines data processing within the surveillance system. For these connections to happen, the database must be built on a Web platform to allow data transfer and communications.

#### Feasibility and Sustainability Attributes

As described previously, replicating MIS modules and front-end functionalities was initially a critical aspect of the Web-based MIS upgrade due to the well-understood challenges of ensuring high quality data from data entry staff. This approach ensured minimal impact to familiarize users with a new system and stayed within the already existing time and financial constraints. In addition, it ensured the MIS upgrade was perceived as a continuation of the previous system rather than a new activity burdening already overstretched workers. These aspects have a significant impact on maintaining sustainability and operation of an already highly accepted system.

In addition, a key principle was to ensure data use and collection at the central level was not interrupted due to a system transition. Hence, the MIS upgrade includes planning for a one-time data migration to be conducted in parallel with the operational capacity for roll-out. This will ensure that data flow will not be diverted into two separate streams on different platforms and databases.

As with the initial MIS development, systems modifications and development are conducted under the leadership of CNM to ensure that new functionalities respond to country needs. This process is particularly important as Cambodia builds from its previous MIS experience and looks to preserve full ownership of the system. Upgrading a preexisting malaria surveillance system while maintaining characteristics specific to the country context is critical to ensure system acceptance and sustainability.

In countries where a functional case-based surveillance system is already developed and operational, with all levels of the health system invested in its usage, building and adapting that existing system to address the technical upgrade needs is the optimal approach. Rather than set up a brand new system on a different platform, modifying the established system also ensures that ownership is maintained with minimal additional training needs. Other neighboring countries in the region that used case-based reporting systems pre-elimination have followed this phased approach to great success [13,14].

Countries in the malaria control phase without a previously functional surveillance or information system typically opt to incorporate a system following a predeveloped data warehouse model. In this scenario, the central database and most core pre-elimination and elimination functionalities are already created and readily available for minor modifications. However, customization and programming remain a challenge to address country-specific contextual factors such as local language support, incorporation of the existing health system structure, and the need for elimination module adaptation to the national program’s operational and procedural decisions. Regardless of the pre-elimination situation, it is still essential to carefully plan a transition phase to ensure that the existing system, whatever its form, can migrate into the new phase with minimal disruption. “One size does not fit all” clearly applies to surveillance and information systems. Despite the pressing global and regional agendas involved with elimination efforts, it is critical that countries undertake a comprehensive needs assessment exercise capturing dimensions for a well-functioning system that go beyond functionality and operationalization, while including feasibility and sustainability attributes to guide country-specific decision making.

## Conclusions

As countries move toward malaria elimination, national programs gather experiences and lessons learned in the process of strengthening their surveillance systems and strategies throughout the control-pre–elimination-elimination continuum. Sharing these experiences not only facilitates the establishment of “best practices” but also accelerates worldwide and regional malaria elimination efforts. Countries at different transitioning stages have the benefit of learning from and incorporating or modifying methods implemented in other countries which have already passed through the same phases. Cambodia’s experience in developing and upgrading its MIS to remain responsive to country-specific needs demonstrates the necessity for considering functionality, operationalization, feasibility, and sustainability of an information system in the context of malaria elimination.

## Appendix

Multimedia Appendix 1 Malaria information system upgrade phasing.

## Abbreviations

CNM: National Centre for Parasitology, Entomology, and Malaria Control
GMS: Greater Mekong Subregion
HC: health center
HF: health facility
HMIS: health management information system
MEAF: malaria elimination action framework 2016-2020
MIS: malaria information system
MOH: ministry of health
OD: operational district
PHD: provincial health department
PPM: public private mix
RACD: reactive case detection
VMW: village malaria worker

## References

[1] World Health Organization (2015). World Malaria Report 2015.

[2] National Center for Entomology, Parasitology and Malaria Control (CNM) (2011). The National Strategic Plan for Elimination of Malaria in the Kingdom of Cambodia 2011-2025.

[3] National Center for Entomology, Parasitology and Malaria Control (CNM) (2016). Cambodia Malaria Elimination Action Framework 2016-2020.

[4] Mellor S (2013). Malaria Consortium.

[5] Ohrt C, Roberts KW, Sturrock HJ, Wegbreit J, Lee BY, Gosling RD (2015). Information systems to support surveillance for malaria elimination. Am J Trop Med Hyg.

[6] Cox J, Sovannaroth S, Dy SL, Ngor P, Mellor S, Roca-Feltrer A (2014). Novel approaches to risk stratification to support malaria elimination: an example from Cambodia. Malar J.

[7] World Health Organization (2012). Disease Surveillance for Malaria Elimination: an operational manual.

[8] Khamsiriwatchara A, Sudathip P, Sawang S, Vijakadge S, Potithavoranan T, Sangvichean A, Satimai W, Delacollette C, Singhasivanon P, Lawpoolsri S, Kaewkungwal J (2012). Artemisinin resistance containment project in Thailand. (I): Implementation of electronic-based malaria information system for early case detection and individual case management in provinces along the Thai-Cambodian border. Malar J.

[9] Barclay VC, Smith RA, Findeis JL (2012). Surveillance considerations for malaria elimination. Malar J.

[10] malERA Consultative Group on Basic ScienceEnabling Technologies (2011). A research agenda for malaria eradication: basic science and enabling technologies. PLoS Med.

[11] Robert Black, World Health Organization (1968). Manual of epidemiology and epidemiological services in malaria programmes.

[12] Chisha Z, Larsen, DA, Burns M, Miller JM, Chirwa J, Mbwili C, Bridges DJ, Kamuliwo M, Hawela M, Tan KR, Craig AS, Winters AM (2015). RESEARCH open access enhanced surveillance and data feedback loop associated with improved malaria data in Lusaka, Zambia. Malaria Journal.

[13] Ma S, Lawpoolsri S, Soonthornworasiri N, Khamsiriwatchara A, Jandee K, Taweeseneepitch K, Pawarana R, Jaiklaew S, Kijsanayotin B, Kaewkungwal J (2016). Effectiveness of implementation of electronic malaria information system as the national malaria surveillance system in Thailand. JMIR Public Health Surveill.

[14] Cao J, Sturrock Hugh J W, Cotter C, Zhou S, Zhou H, Liu Y, Tang L, Gosling RD, Feachem Richard G A, Gao Q (2014). Communicating and monitoring surveillance and response activities for malaria elimination: China's “1-3-7” strategy. PLoS Med.

