# Supplementary material for: Experiences From Developing and Upgrading a Web-Based Surveillance System for Malaria Elimination in Cambodia
Source: JMIR Public Health Surveill. 2017 Jun 14;3(2):e30. doi: 10.2196/publichealth.6942 (PMC5489705; doi:10.2196/publichealth.6942)
Supplement: Multimedia Appendix 1 [file publichealth_v3i2e30_app1.pdf]

Appendix 1 Malaria information system upgrade phasing.

System Upgrade Elements	Areas of implementation	Microsoft Access: local MIS[a] (existing system)	Web-based MIS (phase 1)	Web-based MIS (phase 2)
**Reporting points of care**				
	Elimination OD[b]	VMW[c], HF[d], private providers		
	Burden reduction OD	VMW, HF, private providers		
**Data reporting time and characteristic**				
	Elimination OD	Passive, monthly case-based line list	Passive, monthly case-based line list	Passive, real-time case reporting. Active case finding if defined by surveillance operations
	Burden reduction OD			Passive, monthly case-based line list
**Data entry in the system**				
	Elimination OD	OD staff enters data from paper forms into Access database, then sends to central level	OD staff enters data from paper forms through a Web portal	Individual service providers report data directly at point of care through mobile platforms
	Burden reduction OD			OD staff enters data from paper forms through a Web portal. To be phased out with demonstration of high data accuracy and consistency.

**Platform for data entry**				
	Elimination OD	Local, offline platform (Access).	Web-based platform utilizing cloud server. Data immediately available to all parties granted access.	Case reporting mobile application, customized to point of care source. Data immediately available on a cloud server
	Burden reduction OD			Web-based platform utilizing cloud server. Data immediately available to all parties granted access.
	Elimination OD	Case-based line listing<br><br>Intervention coverage (bed nets only)<br><br>Village Information	Case-based line listing<br>Intervention coverage (bed nets only)<br><br>Village information	Case-based line listing<br>Case investigation<br>Case response<br>Foci investigation<br>Foci response<br>Intervention coverage (bed net and others)<br>Village information
	Burden reduction OD		Case-based line listing<br>Intervention coverage (bed nets only)<br><br>Village information	Case-based line listing<br><br>Intervention coverage (bed net and others)<br><br>Village information

	Elimination OD	Basic coverage indicators (incidence reports, bed nets, and so on).<br><br>Quarterly bulletins<br><br>Stratification reports	Automated epidemiological and geographic reports with key indicators (at OD level). All retrospective data immediately available.<br><br>Case investigation data, including both operational and clinical information. (eg, proportion cases investigated).<br><br>Village risk stratification	Automated epidemiological and geographic reports with key indicators (at OD and HF level). All retrospective data immediately available.<br><br>All surveillance activities' data, including: case investigation and response; Foci investigation and response
	Burden reduction OD		Automated epidemiological and geographic reports with key indicators (at OD level). All retrospective data immediately available.<br><br>Village risk stratification	Automated epidemiological and geographic reports with key indicators (at OD and HF level).<br><br>Village risk stratification

	Elimination OD	Central level (compilation, analysis, and editing).<br><br>OD level (data entry and simple visualization)	Central level (compilation, analysis, editing, and user management).<br><br>OD level (data entry, analysis and visualization)	Central level (compilation, analysis, editing, and user management)<br><br>OD level (data entry, analysis, and visualization)<br><br>Point of care (data entry and operational response communications)
	Burden reduction OD			Central level (compilation, analysis, editing, and user management)<br><br>OD level (data entry, analysis, and visualization)
	Central level	Relational tables separated by reporting source.<br><br>Central source—defined records between tables	Restructured central case table generates framework with unique case identifiers independent of reporting source. Specific case characteristics (localization, clinical data, surveillance info, and so on) added based on available data and operational response.<br><br>Individual cases are functionally linked to their respective surveillance responses.	

		(village, health facility, and OD codes).<br><br>Lack of flexibility to integrate additional post diagnosis case data.	Parity and efficiency in system operations

[a]MIS: malaria information system.

[b]OD: operational district.

[c]VMW: village malaria workers.

[d]HF: health facility.
